# Portable UV-C Device to Treat High Flow of Infectious Aerosols Generated during Clinical Respiratory Care

**DOI:** 10.21203/rs.3.rs-4648863/v1

**Published:** 2024-07-26

**Authors:** Richard Vincent, David Rapoport, Priti Balchandani, Joseph Borrello, Michael Schotsaert, Robert Karlicek, Gabriel Laghlali, Prajakta Warang, Seokchan Park, Gagandeep Singh, Isabella Morgan, James Paredes, Raveen Rathnasinghe, Jacob Wolf, Adolfo Garcia-Sastre

**Affiliations:** Icahn School of Medicine at Mount Sinai; Icahn School of Medicine at Mount Sinai; Icahn School of Medicine at Mount Sinai; Icahn School of Medicine at Mount Sinai; Icahn School of Medicine at Mount Sinai; Rensselaer Polytechnic Institute; Icahn School of Medicine at Mount Sinai; Icahn School of Medicine at Mount Sinai; Icahn School of Medicine at Mount Sinai; Icahn School of Medicine at Mount Sinai; Icahn School of Medicine at Mount Sinai; Independent Consultant; Icahn School of Medicine at Mount Sinai; Icahn School of Medicine at Mount Sinai; Icahn School of Medicine at Mount Sinai

## Abstract

Respiratory interventions including noninvasive ventilation, continuous positive airway pressure and high-flow nasal oxygen generated infectious aerosols may increase risk of airborne disease (SARS-CoV-2, influenza virus) transmission to healthcare workers. We developed/tested a prototype portable UV-C_254_ device to sterilize high flows of viral-contaminated air from a simulated patient source at airflow rates of up to 100 l/m. Our device consisted of a central quartz tube surrounded 6 high-output UV-C_254_ lamps, within a larger cylinder allowing recirculation past the UV-C_254_ lamps a second time before exiting the device. Testing was with nebulized A/PR/8/34 (H1N1) influenza virus. RNA extraction and qRT-PCR showed virus transited through the prototype. Turning on varying numbers of lamps controlled the dose of UVC. Viability experiments at low, medium and high (100 l/min) flows of contaminated gas were conducted with 6, 4, 2 and 1 lamp activated (single-pass and recirculation were tested). Our data show 5-log reduction in particle forming units from a single lamp (single- pass and recirculated conditions) at high and low flows. UVC dose at 100 l/m was calculated at 11.6 mJ/cm^2^ single pass and 104 mJ/cm^2^ recirculated. The protype device shows high efficacy in killing nebulized influenza virus in a high flow of contaminated air.

## Introduction

Since the beginning of the COVID-19 pandemic in March 2020, large numbers of patients throughout the world have presented with highly contagious disease that often includes severe respiratory distress [1]. Treatment in the early days of the pandemic focused on delivering high fractions of inspired O_2_ (FiO2) and mechanical ventilatory support [2–3]. These measures were based on the recommendations for patients with respiratory failure during influenza pandemics [4]. To date the WHO lists these interventions as “aerosol generating procedures,” which implies they carry a significant risk of infecting health care workers (HCW) [5]. Much of this risk seems to have been mitigated by isolation and early adoption of highly effective personal protective equipment (PPE), including gowns and N95 respirators [6]. However, there continues to be debate about how to mitigate contagion risk to HCW and the general public when an infected patient is not isolated and PPE precautions are less than optimal, as in environments where there are limited health care resources, overwhelmed hospital wards, or patient overflow into poorly ventilated areas. These issues were also compounded during the early pandemic by supply chain problems that resulted in limited availability of adequate PPE [7]. The importance of access to PPE motivated an earlier effort by our team to extend the supply of N95 respirators by showing that SARS-CoV-2 inoculated N95 respirator masks could be disinfected by 254 nm wavelength Ultraviolet-C light (UV-C_254_) [8].

The degree to which standard of care procedures used in influenza and COVID-19 respiratory distress increase particulate and aerosol viral spread continues to be studied and debated [8–9]. Highly effective treatments for respiratory distress and failure include intubation and mechanical ventilation, thought to cause an acute risk of aerosol generation during intubation followed by minimal aerosol dispersion due to the closed circuit used by respirators; non-invasive mask ventilation with single circuit gas delivery systems - that require an intentional leak-port as well as employing leak-prone mask interfaces; standard nasal O_2_ and high flow nasal O_2_ – that deliver up to 70 L/min of overflow - may contaminate the air in the room. It has been assumed that all the treatments above, to a variable degree, result in dispersion of exhaled and potentially infectious particles, although recent literature has challenged the degree to which this increases the risk of viral aerosolization over the risk inherent in simple breathing, talking and coughing by a patient [10]. There is, however, general consensus that there is some increase in particle generation with all of the above procedures, which is amenable to controls in contrast to patient behaviors (particularly cough). Thus, some of the therapies can play a role in exposure of HCW to viral particles. Much research, expense, and discussion of resource allocation has been expended to mitigating this. The greatest effort has gone into emphasizing use of PPE, maximizing room ventilation to dilute any exhaled particles, and various approaches at disinfecting room air, as with upper room UV-C_254_ fixtures. [10–11].

While the above approaches are unquestionably effective for reducing accumulated contamination in enclosed spaces, a plume of contaminated gas (as from a cough or at the egress of air from a respiratory treatment device), creates a particularly high, and distinct, risk of contagion, particularly when it experiences a high and directional flow rate.

Two approaches to addressing the exhalate in high flows of contaminated air are in wide use: personal protective, high efficiency filtering masks (N95-N99) and disinfecting the gas stream by UV-C_254_, ultraviolet germicidal irradiation (UVGI). Limitations of N95 filtered masks include cost and the need to replace them often, especially when they are subjected to high humidity (as in high flow nasal O_2_. Non-Invasive Ventilation (NIV) and mask continuous positive airway pressure (CPAP) circuits); this is because filters cease to function well when wet due to reduced filtering efficacy and due to increased resistance [12–13]. Especially during outbreaks of pandemics, stocks of N95 filtered masks may be depleted fast as was the case in the beginning of the COVID-19 pandemic, which left even those at enhanced exposure risk like health care workers unprotected worldwide [14]. Use of UV-C is an attractive anti-viral approach and has long been used to reduce infection in TB units and other medical (and non-medical) environments where aerosols play a role in disease transmission [15]. However, most UV-C devices proposed to date have been for open spaces [16]; these devices are not suitable for rapid action on contained high flows of gas. Because therapies such as high flow nasal O_2_ generate flows of contaminated gas of up to 100 L/min, even when the gas is passed in close proximity to a UV–C source (typically UV-C radiation from a low-pressure mercury vapor (LPMV) lamp at 254 nm) there are only short periods of contact. To overcome this, either a long LPMV lamp emitting UV over its length or a very high UV-C_254_ intensity source is needed. A linear version of the former is impractical, and the latter will generate significant heat that must be dissipated as well as potentially cause unsafe levels of UV in the room.

In view of early recognition of the above needs and limitations of presently available devices, a group was convened in the spring of 2020 based on a proposal by one of the authors (JP) to develop a UV-C_254_ device that would treat gas flowing through a tube at up to 100 L/m, both sterilizing any viral particles in the stream and maximizing dispersion of heat generated. An additional goal was to make this available at a price that would be attractive to low-resource environments (< US$500) and easily commercialized.

We used H1N1 as a viral challenge because mechanical ventilation was used in previous outbreaks of highly pathogenic influenza virus H5N1. [3] The present paper describes our current device and BSL-2 bench tests performed on H1N1 virus to validate its efficacy.

## Materials and Methods

### Design and building of the prototype.

**Figure 1:**
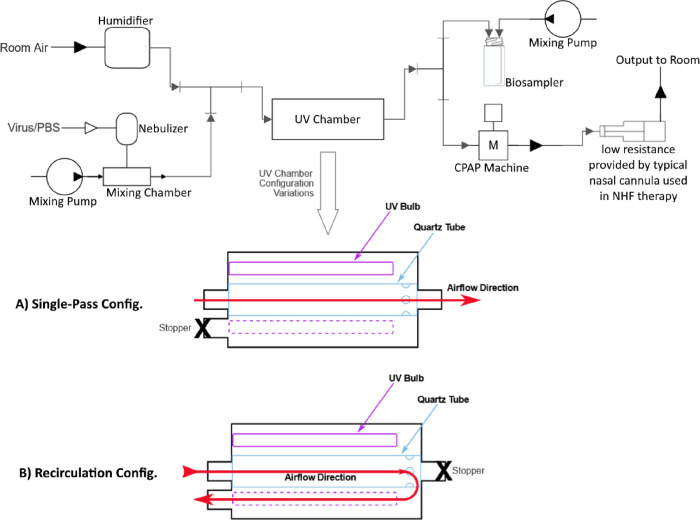
Experimental Test Apparatus with Prototype in the Circuit. Schematic of portable UV-C device. (insert)

The portable UV-C_254_ high-flow aerosol inactivation device was conceived during the COVID-19 pandemic. An inventor (JP) working in collaboration with several departments at the Icahn School of Medicine at Mount Sinai (ISMMS) and Rensselaer Polytechnic Institute (RPI) rapidly modeled a series of CAD designs. From these designs, a cylinder made of a high-UV-C reflective Polytetrafluoroethylene (PTFE) Teflon was fabricated (Altamira Materials Solutions, Houston). A conceptual schematic is shown in Fig. 1 (insert). Air flows into a central quartz tube 50 mm diameter and 40 cm length (transparent to UV-C) surrounded by 6 high-output UV-C_254_ lamps (UV 05–0331-R GPH436T5L/HO/4PSE, Atlantic Ultraviolet, Hauppauge). Each lamp is rated to emit 13 UV watts for an input of 48 electrical watts. At the distal end of the central quartz tube, air recirculates into the larger bore tube for a second pass across the UV-C_254_ lamps before exiting the device. The air path is low resistance and keeps gas in contact with the emitted UV-C radiation for maximal time. The dimensions of the initial (tested) version are 305mm (~ 12”) in length and 127 mm (5”) in diameter. The second pass maximizes contact of the airstream with UV-C and is also intended to maximize cooling of the confined UV-C lamps. To test UV-C dosing, the six high-output UV-C lamps were arranged hexagonally around the central flow tube and could be turned on individually. The combined UV-C output for the six lamps is 78 UV watts 254 nm. Measurements were made of UV flux, with a progressively increasing number of powered lamps made active, using a handheld radiometer (Optometer P9710 with 360°-UC18–2 detector, Gigahertz-Optik, Tuerkenfeld by Munich, Germany).

## Experimental Setup

Our UV-C_254_ prototype was attached to a bioaerosol generating circuit to simulate an infected patient (See Fig. 1). At the beginning of the bioaerosol transmission circuit, a misting nebulizer (Aeroneb Lab Micropump Nebulizer, Kent Scientific, Torrington, CT) was inserted to aerosolize the A/PR/8/34 H1N1 influenza viral suspension of 10^7^ PFU mixed with 10 mL Phosphate Buffer Solution (PBS) into the airstream that entered the tubing connected to the prototype. The misting nebulizer allowed less turbulent nebulization conditions than a previous respiratory nebulizer, which enhanced virus viability at the end of the circuit. One or two parallel standard CPAP generators (Resmed S9, San Diego) were used to create suction as an airflow generator. These provided various flow rates (suction was used rather than positive airway pressure to minimize leaks from the circuit) through the entire aerosol sampling circuit. A setting on the flow generators intended to generate a CPAP pressure of 10 cm H_2_0 generated a high flow when connected to the circuit. The gas was humidified to approximately 100% relative humidity (RH) with distilled water as is typically done in clinical situations of CPAP and nasal NHF (HC 150 Fisher and Paykel Healthcare, New Zealand). This high RH was intended to mimic clinical usage and to keep the viral suspension from desiccation. Various humidification levels were tested for their possible effect on virus viability. The flow rate through the circuit during the experiments was measured (TSI 5300 Series Digital flow meter, TSI, Shoreview, Minnesota) both upstream and downstream of the prototype to identify any leaks in the system. Ambient relative humidity, as well as at the RH at the beginning and end of the circuit, were measured with a calibrated psychrometer (General Tools No. EP8709, China). As the virus contaminated gas exited the prototype (either in single pass or recirculation mode (Fig. 1a & b)) with increasing numbers of UV lamps on or off, it was collected with an SKC Biosampler, 3 nozzles with sonic orifices, (SKC, Inc, Eighty Four, PA). The SKC Biosampler was connected to a SKC BioLite pump to achieve sonic flow within the Biosampler. A separate trap was placed in line after the Biosampler circuit to collect any liquid overflows. Airflow speed through the entire circuit was regulated by changing the setting for CPAP pressure, and ultimately by adding a second parallel CPAP device to increase flow (ResMed, San Diego) depending on the velocity (low, medium, high) to be tested. A HEPA filter was placed in line before the CPAP flow generator to prevent virus from entering the device.

### Virus Stock.

150 plaque forming units (PFU) of the A/PR/8/34 (H1N1) influenza virus were injected into the allantoic fluid of 8-days old embryonated chicken eggs and was incubated for 48 hours at 37°C followed by an overnight incubation at 4°C. Allantoic fluid, containing the virus, was carefully collected, briefly centrifuged, aliquoted and stored at −80°C. The virus stock was titrated by plaque assay on pre-seeded confluent monolayers of Madin-Darbin Canine Kidney (MDCK) cells (ATCC CCL-34).

### Virus titration by plaque assay.

For plaque assays, 250 μL of tenfold dilutions in PBS of collected samples were incubated on confluent monolayers of MDCK cells at 37°C. After 1 hour of incubation, the inoculum was removed by aspiration and cells were overlaid with 2% oxoid agar (Oxoid, Basingstoke, UK) mixed with an equal volume of NaHCO3-buffered 2xMEM supplemented with DEAE/Dextran and TPCK-treated trypsin (1 μg/mL). Cells were incubated for 48 hours at 37°C and 5% CO2. Plaque formation was visualized by staining of cell surfaces after fixation with 4% formaldehyde (5 min at room temperature). For staining, cells were incubated with a 1/1000 dilution of post challenge mouse serum followed by incubation with 1/1000 diluted sheep anti-mouse serum conjugated to horse radish peroxidase (GE Healthcare) and addition of TrueBlue substrate (KPL—Seracare, Milford, MA, USA).

### Nebulizer Suspensions.

H1N1 was suspended in Phosphate Buffer Solution (PBS). The titer of freshly prepared nebulizer suspensions was calculated to be 10^7^ PFU/mL and confirmed by plaque assay titration.

### RNA Extraction and Quantitative Reverse-Transcription Polymerase Chain Reaction (qRT-PCR).

RNA was extracted using the E.Z.N.A Viral RNA kit, according to manufacturer’s protocol. RNA analysis was performed by one step qRT-PCR with the Luna Universal One-Step RT-qPCR Kit (NEB) using PR8-NP primers (forward- GCACGGTCTGCACTCATATTGAG and reverse- GTGTGCTGGATTCTCATTTGGTC). A PdZ-NP plasmid was used as an internal control to assess the copy number of NP RNA.

### Test Procedure

**Figure 2:**
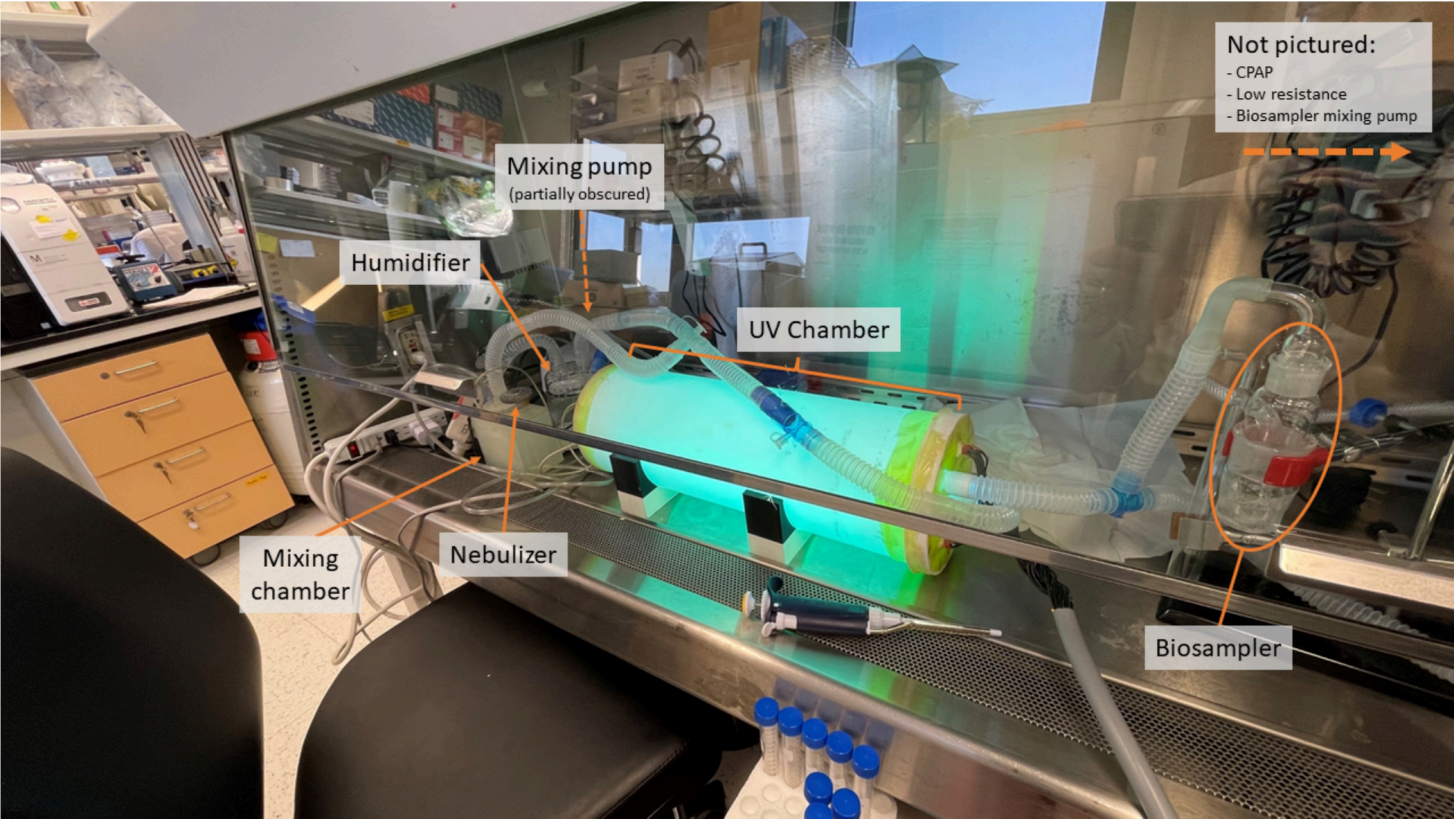
UV-C Prototype Device in test rig within BSL-2 Hood and shown in recirculation condition.

Three rounds of experiments were conducted to determine if, before adding UV irradiation, viable virus could be recovered in sufficient concentration after physical losses within the circuit, after either a single pass or recirculation (Fig. 1a & b) through the prototype device. We conducted separate experiments with nebulized virus at room humidity and at initial relative humidity of 100%. The experiments were conducted at ISMMS in a Biosafety Level 2 (BSL-2) safety hood (Baker Co, Sanford, ME) (Fig. 2). After demonstrating we could culture viable virus from the apparatus, our goal was to provide viral inactivation with gas passing through the portable UV-C_254_ device at up to100 L/min. Our experiments tested various combinations of low (26 L/min), medium (41 L/min) and high (126 L/min) rates of total flow with various combinations of 1 to 6 high-output UV-C lamps switched on. Each experiment ran between 20 to 30 minutes.

## Experiments Rounds 1, 2 and 3

Experiments were conducted to determine the dose of UV needed to kill the H1N1 virus with an ultimate future goal to test the device in the ISMMS BSL-3 against SARS-CoV-2. The H1N1 series of tests were performed sampling our circuit at three sites, 1) simultaneously detecting viral mRNA and viral cultures from a 20- to 30-min collection of gas into the Biosampler, 2) at the outflow of the nebulizer (before UV exposure), and 3) at the exit of the device (after UV exposure). Measurements were made with both ambient RH, no UV and virus, 100% RH no UV and virus to determine the impact of RH on reduction of virus collected in the Biosampler. Additional measurements were performed with fewer UV-C lamps turned on with low, medium, and high airflow to determine the effect of shorter and longer dwell time and lower UV-C dosing on inactivation of the virus. Measurements were made with 1, 2, 4, and 6 UV-C lamps powered, at each level of flow through the system. Table 1 shows the conditions and study parameters.

## Results and Discussion

**Figure 3:**
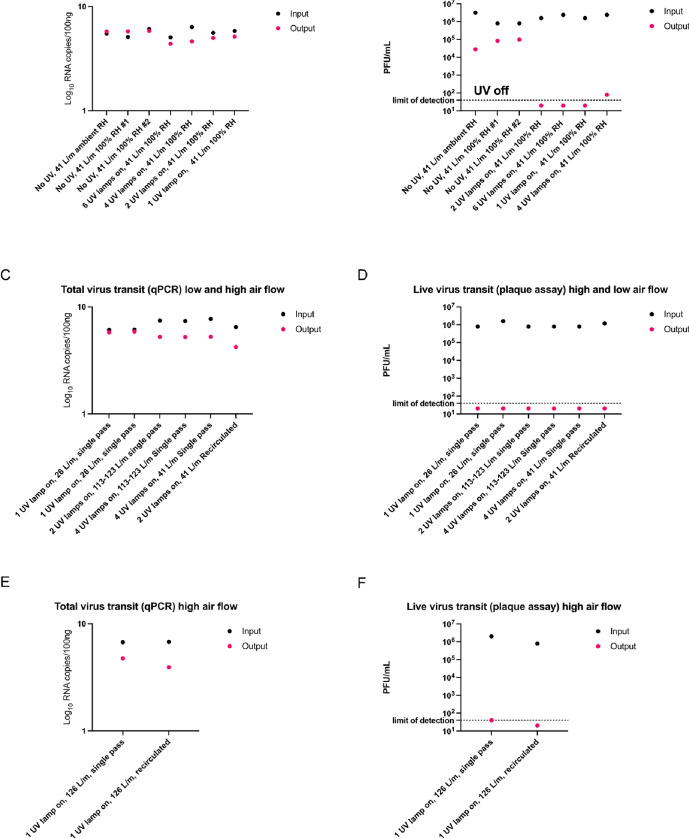
Results of UV-C inactivation of H1N1 virus under different dose, airflow rates and RH paired with mRNA results.

To confirm virus was effectively inactivated by exposure to UV-C light and not, for example by heat or other means (e.g., Influenza virus grows/survives at 37 °C and gets inactivated slowly at higher temperatures), we set up a method to quantify the amount of virus that could be recovered at the outlet of the UV-C device, even when inactivated. Hereto qPCR was performed with primers that detect the influenza virus nucleoprotein (NP) using a plasmid containing the NP gene to calculate gene copy number. H1N1 influenza virus levels were quantified using samples from the input and outlet. This allowed us to confirm that virus was recovered from the device output irrespective of UV treatment or device settings, and that titers showed a reduction up to 2 logs for some of the treatments (Fig 3a, c, e).

### H1N1 Virus UV-C Inactivation

#### Round 1

For single pass-through configuration, (Figure 1 a.) an H1N1viral bioaerosol was aerosolized to move at medium-flow rate, ~41 l/m, in a single pass through our prototype and tested with ambient room humidity (not measured) and humidity added (not measured) at the aerosol generation device to determine viral losses in the circuit without UV-C turned on. The results showed greater consistency with humidity added. Viral inactivation of ~5 log reduction in PFU/ml was shown for all UV-C lamp on conditions either below or at the limit of detection (Figure 3a, b).

#### Round 2

For single pass-through and recirculation configurations (Figure 1 a and b) All conditions during low flow (26 l/m), high flow (113–123 l/m), including with 1, 2 or 4 lamps on and a single pass through the circuit, were below the limit of detection, giving a 5-fold reduction of PFU/ml (Figure 3c, d). At the higher flow (111–133 l/min), with 2 lamps on, using the recirculated circuit, the virus was below the limit of detection for culture (5-fold reduction of PFU/mL).

#### Round 3,

For single pass-through and recirculation configurations (Figure 1 a and b) 1 lamp on, single pass high flow (126 l/m), resulted in 5 log reduction of PFU/ml. 1 lamp on, recirculated high flow (126 l/m), produced a ~5-fold reduction of PFU/ml (Figure 3 e, f).

For Rounds 1, 2 and 3, the H1N1 qPCR data for each Round is shown in Figures 3 a, c, and e respectively.

### TEMPERATURE DATA

We characterized the internal temperature of the prototype with 6, 4, 2, and 1 lamp(s) switched on (Table 2). Temperature was measured in the air gap at the distal end of the prototype, at the middle of the prototype, and at the proximal end of the prototype (where the lamps were plugged into their sockets). Average temperatures were 45°C for 6 lamps, 44°C for 4 lamps, 36°C for 2 lamps and 36°C for 1 lamp.

### UV-C Dose Needed

The prototype device has a volume in contact with the UV-C of 9.62 liters (volume of inner quartz tube .97 l + outer volume recirculation path 8.65 l). At the highest experimental flow rate of 126 l/m (2.1 l/sec), the bioaerosol would spend 0.46 seconds in contact with the UV-C during a single pass and 4.6 sec with the recirculation circuit. With 1 UV-C_254_ lamp producing on average 20 mW/cm^2^ (Table 2) at 36°C, this would be a range of 9.2 mJ/cm^2^ to 82 mJ/cm^2^ which produced a 5 log reduction in PFU/ml. Our design target flow rate is 100 l/m or 1.7 l/sec which would be 0.58 sec for single pass, 5.7 sec for recirculated with a dose range of 11.6 mJ/cm^2^ and 104 mJ/cm^2^.

## Discussion

Our data show that our current prototype inactivates a bioaerosol containing H1N1 virus in large concentrations when exposed to UV-C_254_ with a high flow through the device, consistent with the effluent of several clinically relevant respiratory devices used in treating patients with viral respiratory disease. We confirmed by qPCR that virus was effectively traveling through the UVC device and could be recovered in active or inactivated form at the outlet of the device. Since high concentrations of H1N1 virus could be inactivated with all “lamp on” settings at different flow rates, future designs can take advantage of less or smaller UV-C light sources. The H1N1 influenza virus strain used in these experiments grows optimally at 37°C. Therefore, we assume that the observed temperatures, which were below 37°C on average for some of the settings, will not result in virus inactivation and virus inactivation is due to UV-C exposure. SARS-COV-2 is similarly sensitive to UV-C_254_ [15] and therefore we anticipate that SARS-CoV-2 will be inactivated by similar flows of gas through our protype device, and which will be tested in future experiments. Further refinement of the device is planned with optimization of shape and size, as well as introducing UV LEDs that have recently become available in the range of power needed. With attention to robustness and total cost, we propose that the present approach to sterilizing high flow of contaminated gases has relevance to clinical practice in future pandemics, as well as in the routine management of aerosol generating practices in hospital (and perhaps non-hospital) environments. A recent study by Fisher [17] provided an exposure dose of 21.4 mJ/cm^2^ to block transmission of a bioaerosol of SAR-CoV-2 variants USA-WA1/2020 and Delta between two groups of hamsters. We plan to validate the effectiveness of the next portable device incorporating UVC LEDs first using our BSL2 H1N1 exposure system. We will follow this with experiments in our BSL3 lab using SARS-CoV-2 as the challenge bioaerosol to be inactivated using sentinel hamsters.

## Figures and Tables

**Table 1:** Summary of Conditions for Airflow and UV inactivation Experiments on H1N1.Table 1 Internal Table 2. UV-C and Temperature Readings

**Round 1. Prototype-single pass-through mode (** Fig. 1a **) viral losses from RH, T, airflow, and dose response**
**Viral losses from RH, ambient temperature and moderate airflow q**
**1) Test at medium flow (41 L/m), if losses of initial PFU suspension from ambient room conditions (temperature and humidity, no UV) on survival of virus transported through the entire circuit, prototype and collected in the Biosampler. Test if the PFUs concentrations recovered were sufficient to test UV-C inactivation.**
**2) Repeat the first condition but add 100% RH.**
**3) Repeat condition 2.**
**Viral losses from moderate flow 41 L/m, changing temperature and number of UV-C switched on**
**4) RH 100%, 6 UV-C lamps switched on, temperature from lamps plus cooling of airflow.**
**5) RH 100%, 4 UV-C lamps switched on, temperature from lamps plus cooling of airflow.**
**6) RH 100%, 2 UV-C lamps switched on, temperature from lamps plus cooling of airflow**
**7) RH 100%, 1 UV-C lamp switched on, temperature from lamps plus cooling of airflow.**

Round 2. Single and recirculation modes (Fig. 1a & b)
Prototype single pass-through mode, low and high flow conditions
1) Low flow (26 L/m), humidity added, 1 UV-C lamp switched on
2) Repeat condition 1.
3) High flow (113–123 L/m), humidity added, 1 UV-C lamp switched on
4) High flow (113–123L/m), humidity added, 2 UV-C lamps switched on
5) High flow (113–123 L/m) humidity added, 4 UV-C lamps switched on
Prototype recirculation mode, high flow condition
6) High flow (126–111 L/m), humidity added, 2 UV-C lamps switched on

Round 3 Prototype in high flow single pass and recirculation mode (Fig. 1b)
1) High flow (126 L/m) single pass, 100% RH, 1 UV-C lamp switched on
2) High flow (126 L/m) recirculated, 100% RH, 1 UV-C lamp switched on

## Data Availability

All the data is included in the paper.
